# GROND: a quality-checked and publicly available database of full-length 16S-ITS-23S rRNA operon sequences

**DOI:** 10.1099/mgen.0.001255

**Published:** 2024-06-07

**Authors:** Calum J. Walsh, Meghana Srinivas, Timothy P. Stinear, Douwe van Sinderen, Paul D. Cotter, John G. Kenny

**Affiliations:** 1 Doherty Applied Microbial Genomics, Department of Microbiology & Immunology, The University of Melbourne at the Peter Doherty Institute for Infection & Immunity, 792 Elizabeth Street, Melbourne VIC 3000, Australia; 2 Teagasc Food Research Centre, Moorepark, Cork, Ireland; 3 APC Microbiome Ireland & School of Microbiology, University College Cork, Cork, Ireland; 4 VistaMilk SFI Research Centre, Teagasc Moorepark, Cork, Ireland

**Keywords:** amplicons, database, long read sequencing, microbiome, nanopore, rRNA

## Abstract

Sequence comparison of 16S rRNA PCR amplicons is an established approach to taxonomically identify bacterial isolates and profile complex microbial communities. One potential application of recent advances in long-read sequencing technologies is to sequence entire rRNA operons and capture significantly more phylogenetic information compared to sequencing of the 16S rRNA (or regions thereof) alone, with the potential to increase the proportion of amplicons that can be reliably classified to lower taxonomic ranks. Here we describe *GROND* (*G*enome-derived *R*ibosomal *O*pero*n D*atabase), a publicly available database of quality-checked 16S-ITS-23S rRNA operons, accompanied by multiple taxonomic classifications. *GROND* will aid researchers in analysis of their data and act as a standardised database to allow comparison of results between studies.

**Significance as a BioResource to the community**

DNA sequencing of ribosomal RNA (rRNA) genes, particularly the widely used 16S rRNA gene, plays a pivotal role in bacterial identification and phylogenetic analysis. However, the limitations of traditional Sanger sequencing and short-read sequencing, i.e. low throughput and limited phylogenetic resolution, respectively, have hindered accurate profiling of microbial communities, especially in cases of highly related species. High throughput long-read sequencing technologies from, for example, PacBio and Oxford Nanopore Technologies have revolutionized genome examination capabilities, enabling the sequencing of entire 16S rRNA genes and overcoming these constraints. Here we describe an open-access, quality-checked database containing full-length 16S-ITS-23S rRNA sequences and their associated taxonomy. Extending amplicon-based metagenomic studies to include the 23S gene and ITS region enhances species- and strain-level resolution compared to conventional 16S rRNA sequencing, making it a versatile and valuable resource for researchers wanting to explore microbial diversity at subspecies resolution. This database represents an important resource for the scientific community, facilitating standardized and reliable analysis of microbial communities using long-read sequencing technologies. Its open-access nature promotes general availability and collaboration, allowing researchers to explore and compare results across studies, ultimately advancing our understanding of microbial diversity and evolution.

## Data Summary

The GROND databases, pretrained Naïve-Bayes machine learning classifiers, and statistics describing genome length and *rrn* copy number for each taxon in the databases, are available to download from Zenodo (https://zenodo.org/records/10889037)https://zenodo.org/records/10889037) The code used to generate the databases is available at https://github.com/cazzlewazzle89/GROND.

## Introduction

DNA sequencing of ribosomal RNA (rRNA) genes is an important technique for the identification and phylogenetic analysis of bacteria. Sanger sequencing of the entire ~1.5 kb-encompassing 16S rRNA gene is commonly used to identify cultured isolates and will provide species-level resolution in most cases. However, the 16S rRNA genes of some highly related species, such as members of the *Streptococcus mitis* group or *Escherichia coli* and *Shigella* spp., share >99 % sequence identity and can therefore not be reliably distinguished. Sequencing the longer 23S rRNA gene instead of, or in tandem with, the 16S rRNA gene provides enhanced phylogenetic resolution, thereby allowing reliable separation of these very closely related species. Sanger sequencing is well-established for the purpose of generating long, high fidelity reads from the PCR amplicons that span these rRNA genes. However, Sanger sequencing is inefficient for profiling complex microbial communities and so high-throughput short read sequencing, such as that provided by Illumina, of single hypervariable regions of the 16SrRNA gene is used instead. This approach can generate massive quantities of highly accurate reads but the increased quantity comes with reduced phylogenetic resolution due to substantially shorter read lengths. The 16S rRNA gene has also been the target of choice for amplicon-based metagenomic (a.k.a. metabarcoding) studies due to its mix of alternating highly conserved and hypervariable regions [1,2].

The introduction of long-read sequencing in 16S rRNA gene-targeted metagenomic studies has revolutionised the ability to sequence the entire 16S rRNA gene, overcoming the constraints of short-read technologies, such as Illumina, that were limited to hypervariable regions [3,9]. Pioneered by PacBio and Oxford Nanopore Technologies (ONT), long-read technologies offer the benefits of sequencing long stretches of DNA in a relatively high-throughput, culture-independent, manner. Despite initial challenges with high error rates, both ONT and PacBio have significantly improved their accuracy. ONT’s Q20 +chemistry has notably increased its reliability for 16S rRNA gene studies [10,11], while PacBio has benefited from higher accuracy for a longer period, partly through the application of consensus or HiFi sequencing [12,13]. Additionally, PacBio has made strides in reducing the costs of 16S rRNA sequencing with the introduction of the Kinnex 16S rRNA kit, which utilises the MAS-seq method [14] and their latest sequencing platforms, enhancing the accessibility of full length 16S rRNA sequencing. As the benefits of 16S rRNA gene sequencing become increasingly recognized, the scope of long-read platforms is expanding. They are now being used to sequence both the 16S and 23S genes on a single stretch of DNA while also capturing the internal transcribed spacer (ITS) region. This allows a greater proportion of amplicon sequences to be assigned to species and even strain-level compared to 16S rRNA sequencing [15]. Recent studies have applied this approach to profiling microbial communities [16,17], though they had to rely on custom-built or commercial databases such as Intus Biosciences (formerly Shoreline Biome), which do not allow the direct comparison of results between studies. Recently, others have highlighted the requirement for a regularly updated and publicly available database to format the *rrn* structure for amplicon-mediated metagenomic analyses [18].

Here we present *GROND* (*G*enome-derived *R*ibosomal *O*pero*n D*atabase), an open access quality-checked database of full-length 16S-ITS-23S rRNA sequences, accompanied by taxonomic and contextual data, to act as a database to standardise analysis between studies in the same way silva [19], RDP [20], and Greengenes [21] function for single rRNA genes.

## Database construction

**Fig. 1.**
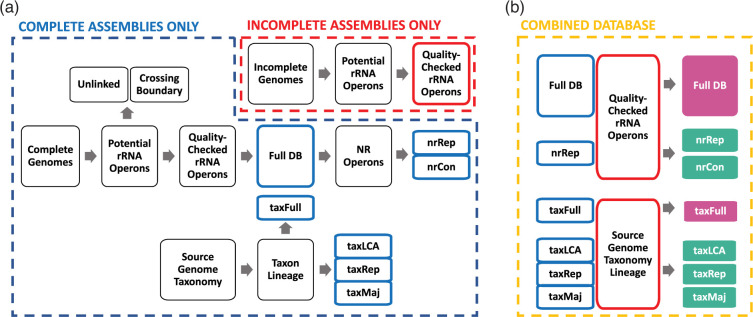
GROND database construction pipeline. Panel (a) depicts intermediary steps and files which are combined and/or dereplicated to generate the final database depicted in Panel (b) (more details in DATABASE CONSTRUCTION section). Colour-outlined rectangles represent intermediary files used to build the colour-filled sequence and taxonomy files available for download.

Two datasets were used as the basis for the *GROND* database, differing primarily by the taxonomy systems employed. The first of these, referred to as *RefSeq* throughout this manuscript, was constructed from all 253 840 RefSeq genome assemblies marked ‘Latest’ on 14 July 2022. The second, referred to as *GTDB*, was constructed from all 317 541 genomes included in the most recent release (07-RS207) of the GTDB database [22]. The pipeline described below, and illustrated in Fig. 1, was used to first construct databases of quality-checked 16S-ITS-23S rRNA operon (*rrn*) sequences from complete genomes and then expand this with sequences from incomplete assemblies, thereby capturing as much diversity as possible while placing a premium on genome assembly quality. The associated annotation information of *RefSeq* and *GTDB* source genomes was downloaded in GFF3 format and rRNA gene features were extracted. If annotations were not available, rRNA genes were identified using barrnap (v 0.9) with default parameters except genes with a coverage of <80 % were marked ‘partial’. The combined annotation information from complete assemblies was imported into R [23] using the *read.gff* function from the *ape* package [24], partial genes were discarded, and *rrn* sequences were identified by iteratively associating 16S genes with their neighbouring 23S gene if it was:

1. Located on the same assembled sequence (contig or scaffold),
2. Encoded on the same strand,
3. Encoded in the order 16S-ITS-23S rRNA.

The potential *rrn* operons identified based on these criteria were further filtered to remove ‘unlinked’ operons with ITS regions longer than 1.5kbp [25] and operons that crossed the start/end boundary of the contig or scaffold, to leave a final dataset of quality-checked *rrn* sequences. As previously reported [25], unlinked *rrn* operons were highly prevalent in the Deinococcus-Thermus and Planctomycetes phyla (Table S1, available in the online version of this article). Each sequence in the final dataset was assigned a unique operon identifier and their nucleotide sequences were written to a single multifasta file using BEDTools [26]. To reduce computation time and aid analysis, a non-redundant (*NR 99.9 %*) database was created for each dataset. First, a multifasta file of high-quality *rrn* operon sequences recovered from complete genomes was sorted by sequence length using BBTools *sortbyname.sh* [27] and clustered based on 99.9 % nucleotide identity using vsearch *--cluster_smallmem* [28]. First sorting by length ensured that the longest sequence in each cluster was retained as the representative sequence. The high-quality *rrn* operon sequences from incomplete assemblies were then appended to the representative sequence multifasta file and re-clustered, meaning that operon sequences from incomplete assemblies were only retained if they expanded on the sequence diversity of sequences from complete genomes. Consensus sequences for each NR cluster were generated using the --consout option. We believe that most users would benefit from using the NR 99.9 % databases built from both complete and incomplete assemblies to maximise phylogenetic range while minimising computation burden. These are available for download as either representative (nrRep) or consensus (nrCon) sequences.

## Database description

**Fig. 2.**
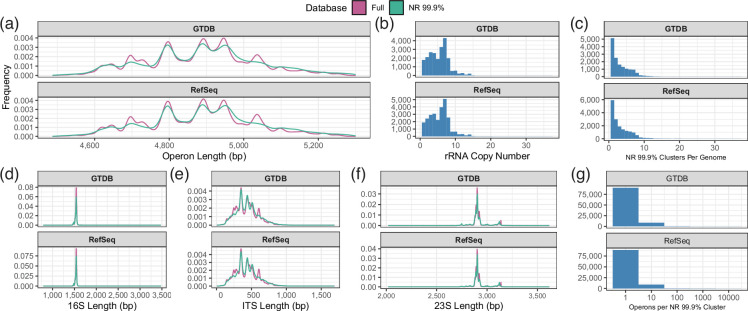
Summary statistics of the full and non-redundant (NR 99.9 %) GROND databases. (**a**) Length distribution of 16S-ITS-23S rRNA operons using the GTDB and RefSeq derived databases. (**b**) Number of distinct rRNA operons in genomes represented in the database. (**c**) Number of NR clusters per genome. **(d–f**) Length distribution of individual 16S-ITS-23S rRNA operon regions. (**g**) Number of rRNA operons represented by each NR cluster. Operons identified as outliers based on length (quartile 1/3±1.5 × interquartile range [IQR]) are not included in this plot to increase readability.

The median length of *rrn* sequences ranged between 4892 and 4899 bp depending on database version (Fig. 2a, Table 1). Of the three constituent regions, the ITS exhibited the greatest length variability across all database versions, followed by the 23S and 16S genes (Fig. 2d–f). When considering complete genomes only, the corresponding source genomes have a mean per-genome rRNA copy number of 5.29 (*s*=2.76) and 5.41 (*s*=2.77) for the GTDB and RefSeq datasets, respectively (Fig. 2b), while 63.62 % or 63.80 % of these genomes were represented in more than one NR cluster (Fig. 2c), supporting previous reports of intragenomic diversity in rRNA genes and ITS regions [29,31]. This is explored further below. For both datasets, approximately 81 % of NR clusters contained just a single *rrn* sequence (Fig. 2g). Dereplication of sequences at 99.9 % identity reduced the GTDB dataset from 291 365 sequences to 103 991, a 64.3 % reduction, and reduced the RefSeq dataset from 317 986 sequences to 103 293, a 67.5 % reduction. Comparatively, this same dereplication was applied to the 16S gene from these *rrn* operon sequences, yielding 58 531 and 55 193 sequences from the GTDB and RefSeq datasets respectively – an approximately two-fold greater reduction.

**Table 1.** Summary statistics describing length (bp) of *rrn* operons and constituent regions for each dataset and database. SD=Standard Deviation, CV=Coefficient of Variation

Dataset	Database	Region	Mean	Median	SD	CV
GTDB	Full	16S	1534.91	1539	30.22	1.97
		23S	2924.31	2903	97.91	3.35
		ITS	439.85	427	182.56	41.51
		*rrn*	4899.05	4893	186.64	3.81
	NR 99.9 %	16S	1532.32	1539	37.21	2.43
		23S	2924.54	2903	117.71	4.02
		ITS	452.85	435	186.57	41.2
		*rrn*	4909.69	4895	205.98	4.2
RefSeq	Full	16S	1536.3	1540	27.16	1.77
		23S	2924.58	2903	90.61	3.1
		ITS	437.56	428	179.54	41.03
		*rrn*	4898.43	4892	179.92	3.67
	NR 99.9 %	16S	1534.44	1539	32.9	2.14
		23S	2927.21	2903	109.82	3.75
		ITS	450.6	436	186.45	41.38
		*rrn*	4912.25	4899	197.58	4.02

## Taxonomy

Taxonomy was assigned to each operon based on the source genome in seven-level format (Kingdom>Phylum>Class >Order>Family>Genus>Species). GTDB taxonomy was readily available to download in this format for archaea and bacteria. RefSeq TaxIDs were converted to this format by TaxonKit [32].

We found that 99.66 % of GTDB NR clusters (and 97.18 % of RefSeq clusters) exhibit 1 % taxonomic agreement in the species-level taxonomy of their constituent operon sequences. In an effort to account for the absence of taxonomic agreement among the remaining clusters, three methods are used to assign taxonomy.

1. taxRep: source genome taxonomy of the cluster representative sequence,
2. taxLCA: lowest common ancestor of all sequences in the cluster,
3. taxMaj: lowest taxonomic rank at which there is a simple majority agreement of all sequences in the cluster.

**Fig. 3.**
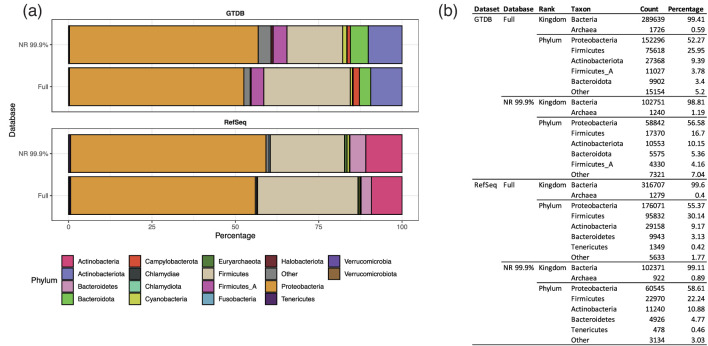
Taxonomic profile of GROND database. (**a**) Phylum-level composition of full and NR 99.9 % databases using GTDB and RefSeq databases and taxonomy systems. (**b**) Kingdom and Phylum-level composition expressed as number of operons and percentage of total.

Files describing these taxonomy systems are available for download with the NR database and phylum-level descriptions are provided in Fig. 3. For most analyses, we recommend the taxRep system. The taxLCA and taxMaj systems are provided to compensate for clusters representing species with unclear or ‘disputed’ taxonomy that may have arisen from incorrect taxonomic assignment of the genome when uploaded to the RefSeq database or an imperfect understanding of what defines a species. For example, a cluster which contains 97 sequences from *E. coli* and three sequences from the *Shigella* genus, two genera which are historically separated but should not be distinct based on genome-level comparative analysis [33], would be classified as s__Escherichia coli by taxMaj, but as f_Enterobacteriaceae by taxLCA.

## Intragenomic diversity

Intragenomic diversity of the *rrn* operon, and the individual constituent 16S, ITS, and 23S regions, was assessed to better understand the diversity of sequences expected for genome identification and microbiome profiling. This was performed on complete GTDB genomes based on the within-cluster taxonomic consistency observed above. From each genome containing more than one *rrn* operon: the regions being compared (*rrn*, 16S, ITS, or 23S) were written to a single multifasta file by BEDtools, a multiple sequence alignment was constructed by MAFFT [34], before being converted to a pairwise distance matrix by EMBOSS *distmat* [35] with option *-nucmethod* 0. These pairwise distance matrices were then imported into R using the *read_phylip_distmat* function in the tidygenomes library [36], where summary statistics and plots were generated using the *tidyverse* [37] and *ggcorrplot* [38] libraries. Pairwise distances are reported by *distmat* as SNVs per 100 nucleotides, so pairwise identities were calculated by subtracting the distances from 100.

**Fig. 4.**
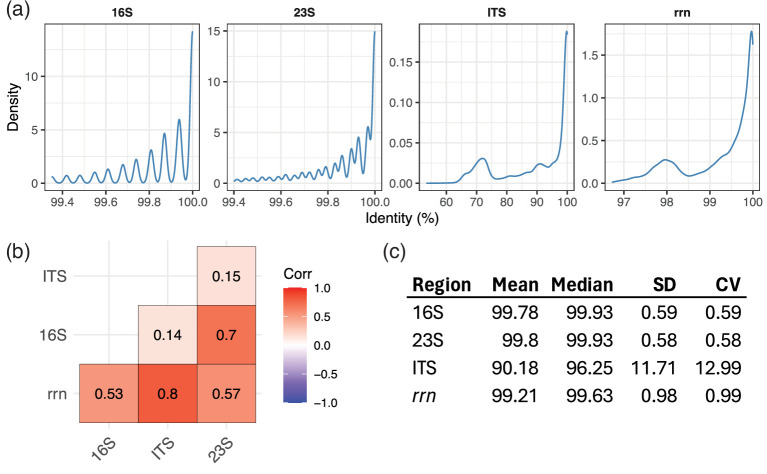
Intragenomic diversity of *rrn* operons and constituent regions from complete GTDB genomes. Plots are based on nucleotide identity between all pairwise intragenomic combinations of *rrn* operons and constituent regions. (**a**) Distribution of pairwise identities. Regions identified as outliers based on length (quartile 1/3±1.5 × interquartile range [IQR]) are not included in this plot to increase readability. (**b**) Pearson correlation of pairwise identities between regions from the same pairwise operon comparisons. (**c**) Summary statistics of intragenomic pairwise identities for the *rrn* operon and the constituent regions. SD=standard deviation, CV=coefficient of variation.

Of the 21 242 complete genomes analysed, 16 680 (78.5 %) exhibited intragenomic diversity of the *rrn* operon, compared to 14 611 (68.8 %), 13 943 (65.6 %), and 13 276 (62.5 %) for the ITS, 23S, and 16S regions respectively. Pairwise intragenomic distances of the entire *rrn* operon followed a roughly bimodal distribution (Fig. 4a), with the major peak at ~99.92 % sequence identity and the minor peak at ~98 % sequence identity, the latter representing ~98 SNVs based on the mean operon sequence length of 4899 bp. This pattern was mirrored by the ITS region which also followed a roughly bimodal distribution, with the major peak at 100 % sequence identity and the minor peak at ~72 % sequence identity, the latter representing ~123 SNVs based on the mean ITS length of 440 bp. This suggests that the main factor driving *rrn* operon intragenomic diversity are variations in the ITS regions, supported by correlation analysis showing a strong (*ρ*=0.8) relationship between intragenomic diversity of the *rrn* and ITS (Fig. 4b). The 16S and 23S genes exhibited similar intragenomic similarity distributions and summary statistics (Fig. 4c). Their diversities were also highly correlated, meaning genomes with higher 16S diversity tended to have higher 23S diversity, and vice versa. Information on the intragenomic diversity of all taxa with complete GTDB genomes is included in GROND.

Similar to previous reports examining intragenomic diversity of the 16S gene [31], we observed that the highest degree of intragenomic *rrn* diversity tended to be present in extremophiles. For example, the halophilic family Haloarculaceae, the psychrophilic genus *Acerihabitans*, and the thermophilic genus *Thermoanaerobacter* all possessed mean *rrn* nucleotide diversities>3 %.

## PCR primer evaluation for amplicon generation

To assess the impact of primer choice on the functionality of the database, we tested three different primer combinations *in silico* (Table 2) using the perl script *in_silico_pcr.pl* (https://github.com/egonozer/in_silico_pcr) to evaluate their amplicon generation efficiency, phylogenetic bias, and amplicon length distribution. Two primer pairs from previous studies focused on the full-length rRNA operon were evaluated, in addition to a new pair that combines the forward and reverse primers from each pair to potentially generate a longer amplicon.

**Table 2.** Primer sequences used for *in silico* PCR analysis

Primer pair	Fwd	Rev	Reference
16S:27 F-23S:2241R	AGRGTTTGATYHTGGCTCAG	ACCRCCCCAGTHAAACT	[45]
16S:27 F-23S:2428R	AGRGTTTGATYHTGGCTCAG	CCRAMCTGTCTCACGACG	This study
16S:519 F-23S:2428R	CAGCMGCCGCGGTAA	CCRAMCTGTCTCACGACG	[17]

**Fig. 5.**
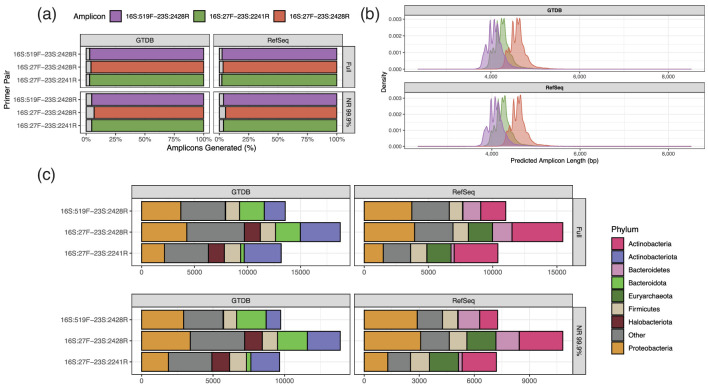
Predicted amplicon generation statistics. (**a**) Percentage of database sequences which were predicted to generate an amplicon by each primer pair. (**b**) Predicted length distribution of amplicons generated from the NR 99.9 % GROND database by each primer pair. (**c**) Phylum-level composition of GROND database sequences where an amplicon was not predicted to be generated by each primer pair.

Based on the predicted primer binding characteristics there is a strong inverse relationship between predicted amplicon length and database coverage meaning there is a trade-off to be considered between phylogenetic range and resolution when selecting primers (Fig. 5a, b). The primer pair 16S:27 F-23S:2428R, which generates the largest amplicons, is predicted to be 1–2 % less sensitive than the other two pairs (Fig. 5a and Table S2).

Primer binding biases were predicted to be relatively consistent between primer pairs at phylum level (Fig. 5c).

## Outlook

We predict that the future of rRNA-based phylogenetic analysis will become increasingly dependent on long-read sequencing technologies due to their superior discriminatory ability. Advancements in sequencing accuracy achieved through ONT R10.4 flow cells [10,11, 39] and PacBio HiFi reads [12,13] have played a pivotal role in this shift from short-read to long-read sequencing. This progression has notably facilitated the rise of full-length 16S rRNA sequencing in recent times. Consequently, phylogenetic analysis based on full-length 16S rRNA currently benefits from well-established laboratory and computational workflows. Meanwhile, *rrn* sequencing is gaining attention for its enhanced resolution in distinguishing closely related species compared to full-length 16S rRNA sequencing [40,44]. However, as *rrn* sequencing is still developing, more advancements and validation are needed before it becomes broadly adopted.

One such advance, essential for the success of any microbiome study, is the construction of reliable databases. The current state of *rrn* sequencing relies on custom-built [16,45,47] or commercial databases [48,49], which results in a lack of standardisation across the field. Moreover, these databases require ongoing updates and maintenance to remain relevant [18]. With these considerations in mind, GROND has been developed to address these shortcomings in *rrn* sequencing. As the most comprehensive *rrn* database to date, and incorporating a GTDB-based version that includes numerous metagenome-assembled genomes (MAGs), this database should prove to be a valuable resource for the *rrn* sequencing community. Indeed, the preprint version of this database has already been used to study bacterial transmission in low-biomass human milk samples [50].

Another key advancement in the adoption of *rrn* sequencing is the standardisation of PCR primers. Variations in microbial profiles have previously been reported depending on the primer pairs used to generate *rrn* amplicons. Kinoshita *et al.* [16] and Martijn *et al.* [17] have demonstrated that the 519 F-2428R primer pair reveals a broader diversity of bacteria and archaea. As a result, while most existing *rrn* databases feature sequences extracted using the 27 F-2241R primer pair [18,46], GROND is intentionally untrimmed so as to be useable by all studies regardless of primer choice, enabling more levelled comparisons in future studies of primer biases in *rrn* sequencing.

The choice of classifier for phylogenetic analysis is significantly influenced by the type of long-read platform employed. The high accuracy of PacBio sequencing data makes it compatible with denoising or clustering pipelines, such as DADA2 [50,51] and vsearch [17,28], respectively. Meanwhile, the lower read quality from ONT demands specialised pipelines to account for its higher error rates. Such pipelines include, alignment using Minimap2 [18,45, 46, 52] or EMU [39,40, 53], or clustering through NanoCLUST [54] or Natrix2 [55]. The application of Unique Molecular Identifiers (UMIs) has shown promise in achieving more accurate ASVs or 97 % OTUs than NanoCLUST-based methods [11,15]. However, this strategy needs further evaluation before it can be applied in *rrn* sequencing for a range of complex communities. The single-nucleotide resolution offered by PacBio HiFi sequencing and UMI-supported ONT sequencing is important as the scope of *rrn* sequencing is widening to include strain-level profiling of microbial communities [48,56, 57], where PacBio’s higher sequencing quality has currently made it the more suitable choice for strain-level detection [12,48, 58].

As we move towards achieving strain-level distinction through *rrn* sequencing, acknowledging the influence of intragenomic variation becomes essential. Bacterial and archaeal genomes typically feature multiple copies of the *rrn* operon and the sequence variation among these copies within a single genome may lead to strain-level misassignments and inflated species-level abundance figures [12,58,60]. It is therefore critical to factor in these intragenomic variations. Sequencing the *rrn* operon, rather than full-length 16S rRNA gene, has proven to more precisely capture the heterogeneity among these copies [12,60]. Techniques based on OTU clustering, rather than exact sequence variants (ESVs), offer a superior way to accommodate intragenomic variation, resulting in more accurate taxonomic profiling when high resolution is necessary [58]. Appropriate clustering thresholds have been established for human microbiome samples specifically for the 16S rRNA gene. However, research on these thresholds for environmental samples and *rrn* sequencing methods is still lacking, presenting an opportunity for further exploration and advancement. Here we have analysed intragenomic diversity of the *rrn* operon and its constituent regions using only complete genome assemblies and operon sequences which passed strict quality control thresholds. However, there are still some factors that must be considered when interpreting these results. First, estimating true copy number can be problematic as these are essentially repetitive regions, which may be collapsed into a smaller number of copies by short-read assemblers. Second, since these repeats may be non-exact, the sequences may be fragmented across multiple contigs. Third, minority variants may not be represented because the assembly algorithm thinks they are errors and ‘corrects’ them, for example if a given genome contains seven copies of the rRNA operon, and at a particular position six have G and one has C, the resulting assembly might lose the C variant and only return the G variants. Using complete genomes for this analysis likely negates the first and second issues but the third may still be present.

As the field of *rrn* sequencing progresses, we can anticipate improved resolution, wider application, and early adoption of standardised methods such as those currently recommended by the Earth Microbiome Project [61]. The GROND database will additionally support this advancement through continual updates with each major RefSeq and GTDB release to keep pace with the ever-growing collection of genome sequences and constantly evolving taxonomy systems.

## supplementary material

10.1099/mgen.0.001255 Uncited Table S1.

## Abbreviations

CoV: coefficient of variation
ESV: exact sequence variants
GROND: genome-derived ribosomal operon database
GTDB: genome taxonomy database
IQR: interquartile range
ITS: internal transcribed spacer
NR: non-redundant
ONT: Oxford Nanopore Technologies
rrn: 16S-ITS-23S rRNA operon
rRNA: ribosomal RNA
SD: standard deviation
taxLCA: lowest common ancestor of all sequences in the cluster
taxMaj: lowest taxonomic rank at which there is a simple majority agreement of all sequences in the cluster
taxRep: source genome taxonomy of the cluster representative sequence,
UMIs: unique molecular identifiers
