# Supplementary material for: GROND: a quality-checked and publicly available database of full-length 16S-ITS-23S rRNA operon sequences
Source: Microb Genom. 2024 Jun 7;10(6):001255. doi: 10.1099/mgen.0.001255 (PMC11261877; doi:10.1099/mgen.0.001255)
Supplement: Uncited Table S1. [file mgen-10-01255-s001.pdf]

Supplementary Material

Table S1. Phylum-level distribution of genomes containing at least one unlinked rrn sequence. RefSeq genome assemblies and taxonomy labels are used and only phyla with> 10 genomes are shown.

**Phylum**	**# Genomes**	**% Genomes Containing Unlinked *rrn* Operons**
Deinococcus-Thermus	73	76.71
Candidatus Thermoplasmatota	14	50
Planctomycetes	57	42.11
Spirochaetes	234	33.33
Proteobacteria	16221	2.82
Crenarchaeota	95	2.11
Firmicutes	6236	0.55
Bacteroidetes	1026	0.49
Actinobacteria	2689	0.26
Tenericutes	452	0.22

Table S2. In silico prediction of primer sensitivity. Sensitivity is expressed as the percentage of sequences in the database predicted to generate an amplicon using each primer pair.

Dataset	Database	Primer Pair	Sensitivity (%)
GTDB	Full	16S:27F-23S:2241R	97.13417878
		16S:27F-23S:2428R	95.94872411
		16S:519F-23S:2428R	97.01714345
	NR 99.9%	16S:27F-23S:2241R	95.35632891
		16S:27F-23S:2428R	93.31769095
		16S:519F-23S:2428R	95.32171053
RefSeq	Full	16S:27F-23S:2241R	97.85839628
		16S:27F-23S:2428R	96.83854006
		16S:519F-23S:2428R	97.67725623
	NR 99.9%	16S:27F-23S:2241R	96.51476867
		16S:27F-23S:2428R	94.7624718
		16S:519F-23S:2428R	96.48185259
